# Nuclear factor kappa B activation in cardiomyocytes by serum of children with obstructive sleep apnea syndrome

**DOI:** 10.1038/s41598-020-79187-0

**Published:** 2020-12-17

**Authors:** Aviv D. Goldbart, Meital Gannot, Hen Haddad, Jacob Gopas

**Affiliations:** 1 Department of Pediatrics, Faculty of Health Sciences, Soroka University Medical Center, Ben-Gurion University of the Negev, P.O.B. 151, 84101 Beer Sheva, Israel; 2 Pediatric Pulmonary and Sleep Research Laboratory, Faculty of Health Sciences, Soroka University Medical Center, Ben-Gurion University of the Negev, Beer Sheva, Israel; 3 Department of Microbiology, Immunology and Genetics, Faculty of Health Sciences, Soroka University Medical Center, Ben-Gurion University of the Negev, Beer Sheva, Israel

**Keywords:** Paediatric research, Experimental models of disease

## Abstract

Obstructive sleep apnea syndrome (OSA) is associated with cardiovascular morbidity in adults and children. NFκB activity is enhanced in circulating monocytes of adults with OSA, that decreases following positive pressure therapy. OSA children’s serum activates NFκB in a cell line. We hypothesized that OSA children’s serum can activate NFκB in cardiomyocytes (CM) and effect their viability. In order to explore the role played by NFκB in OSA cardiovascular pathophysiology, rat, mouse and human immortalized CM were exposed to human serum drawn from OSA children and matched controls. Increased expression of NFκB classical subunits p65/p50 as well as major morphological changes occurred in cardiomyocytes following OSA’s serum exposure. OSA children’s serum induced NFκB activity as measured by p65 nuclear translocation in immortalized human CM and rat cardiomyocytes as well as dense immunostaining of the nucleus. Trypan blue and XTT assays showed that OSA sera induced CM apoptosis. We conclude that NFκB is systemically activated in cardiomyocytes, who also demonstrate decreased viability and contractility following exposure to OSA serum. It supports the hypothesis NFκB plays a role in the evolution of cardiovascular morbidity in OSA. It may support the search for new therapeutic interventions controlling NFκB activation in OSA.

## Introduction

Obstructive sleep apnea (OSA) is a prevalent disorder in children (2–3%)^1^, and it is associated with cardiovascular morbidity^2,3^. Children with moderate to severe OSA, as defined by polysomnography are usually treated with Adenotonsillectomy (T&A)^4,5^. In adults, this apparently simple mechanical disorder is commonly associated with inflammation of the nasopharynx and oropharynx as well as systemic inflammation^6^. NF-κB Activation and elevation of its dependent genes such as TNF-alpha, constitutes an important pathway connecting OSA with systemic inflammation and end organ cardiovascular disease^7^. In adults, NF-κB activity is increased in circulating neutrophils and monocytes, and decreases following conventional l therapy with continuous positive airway pressure (CPAP)^8,9^. Systemic inflammatory markers like C-reactive protein, a known cardiovascular risk marker, are increased in children with OSAS and decreases following T&A^10,11^. The nuclear factor κB (NF-κB) family of transcription factors has a key role in regulation of immune responses, proliferation, apoptosis and expression of certain viral genes^12^, as well as in childhood chronic inflammatory disorders like inflammatory bowel disease^13^, and asthma^14^. Therefore, the NF-κB signaling pathway is considered a target for pharmacological intervention. The therapeutic and preventive effects of many natural products may, at least in part, be due to their capability to inhibit NF-κB^15^. The two known pathways for NF-κB activation are the canonical (classical) and the non-canonical (alternative) pathways. A functional NF-κB molecule is a homo/heterodimer composed of members of the Rel family of proteins. The family includes RelA (p65) and p50 in the classical pathway and RelB, cRel and p52 in alternative pathway. NF-κB is preserved in an inactive form in the cytoplasm by the inhibitor IκB, which binds to NF-κB and masks its nuclear localization signal^16^. IκB is phosphorylated by the IκB kinase complex (IKK), subsequently degraded by the proteosome releases NF-κB. NF-κB then translocates to the nucleus where it stimulates the transcription of a wide spectrum of genes, and activates an inflammatory response.

Because children with OSA present CV morbidity and they demonstrate systemic inflammatory processes, led us to hypothesize that particular circulating inflammatory mediators, in children with OSA can affect cardiomyocytes.

We therefore investigated the effects of serum of children with OSA on NF-κB pathway in cardiomyocytes from several sources.

## Results

### OSA and non OSA patients’ sera

We recruited 30 children to this study (15 OSA and 15 non OSA). Samples were drawn at the operating room, right before the child underwent tonsillectomy. Children were operated 3.1 ± 1.2 months (range 2.3–6.2 m) months following their polysomnographic study. The important characteristics of the children are summarized at Table 1.

**Table 1:** Antropometric and polysomnographic main charachteristics of the 15 OSA and 15 non OSA pt.

	OSA	Non OSA	P value
Patients (N)	15	15	
Mean age (years)	5.0 ± 3.1 (range 3.2–8.1)	5.2 ± 3.4 (range 3.1–9.3)	NS (0.83)
F/ M (n)	6/9	7/8	NS (0.79)
BMI (z score)	0.60 ± 1.01	0.59 ± 1.07	NS (0.87)
AHI (/h TST)	16.1 ± 2.7 (range 10.0–17.4)	0.7 ± 0.3 (range 0.3–0.9)	0.0004

*AHI* Apnea Hypopnea Index, *TST* total sleep time, *NS* not significant.

### P65 and P50 are over expressed by stimulation of OSA patients’ sera

**Figure 1:**
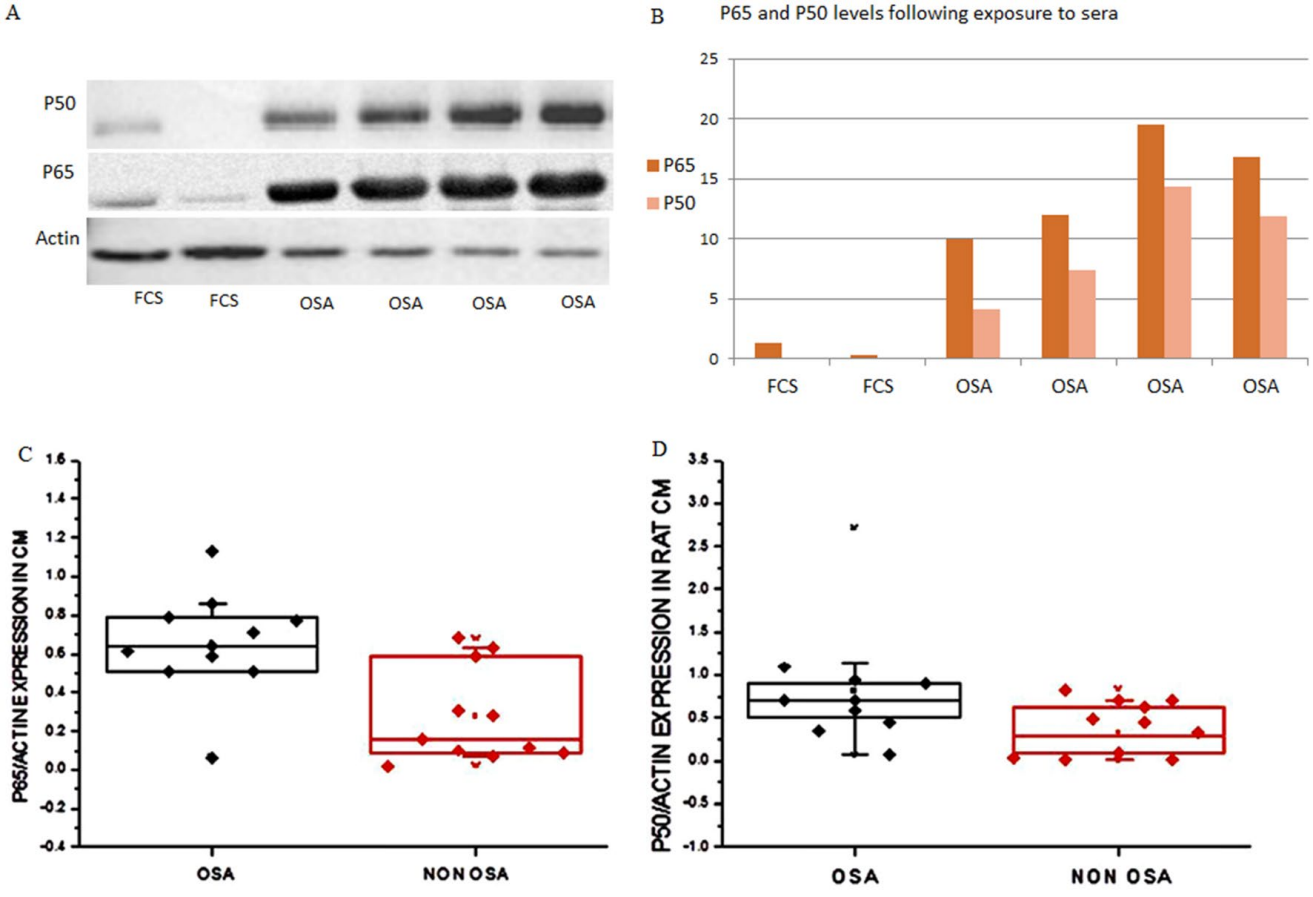
(**A**) Western blot of NFκB sub-units P65 and P50 in whole cell lysates prepared from neonatal rat CM following 2 h exposure to OSA sera or FCS. (**B**) Densitometry of the western blot shown in (**A**). Bands were normalized to their respective actin bands. CM were incubated with 5% OSA or FCS in M-119 medium. Statistical distribution of (**C**) P65 and (**D**) P50 expression levels in whole cell lysates prepared from neonatal rat CM exposed to OSA (black) or non-OSA (red) sera. Horizontal lines represent median values; the upper and lower box limits indicate the 25th percentile and 75th percentile; whiskers represent the 10th and 90th percentiles.

Firstly, we assessed the effect of OSA sera on NFκB sub-units P50 and P65 in rat CM. We exposed cultured CM to different samples of OSA sera or FCS for 2 h, and quantified the expression via western blots. We also exposed rat CM to OSA and non-OSA sera different samples. In Both experiments both subunits were significantly overexpressed at the CM exposed to the OSA sera vs. those exposed to non-OSA or to FCS (Fig. 1).

### NFκB is activated by stimulation with OSA patients sera

**Figure 2:**
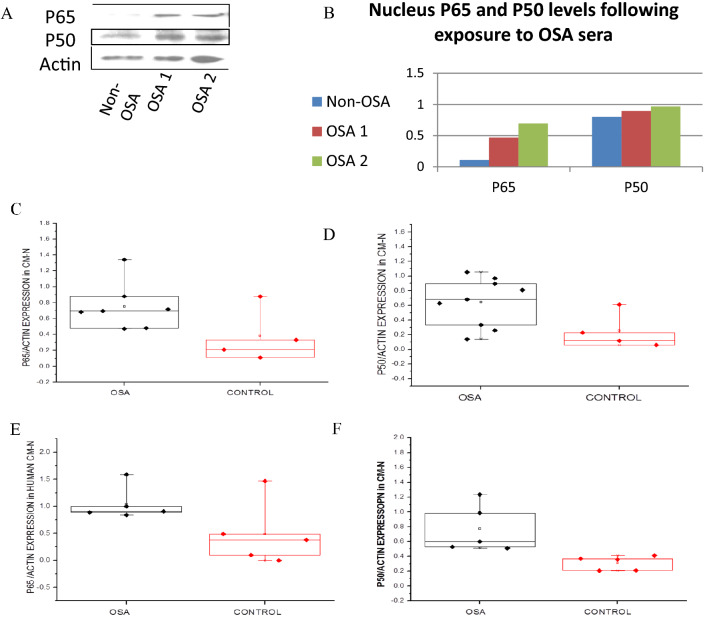
(**A**) Western blot of NFκB sub-units (P65, P50) in rat CM nuclear phase lysates following 2 h exposure to OSA or non-OSA sera. “Non-OSA” refers to 5% non-OSA patient serum; “OSA” refers to 5% severe OSA patient serum, both in M-119 medium. (**B**) Western blot densitometry. NFκB bands were normalized to their respective actin bands. Statistical distribution of (**C**) P65 and (**D**) P50 levels of nucleus lysates prepared from neonatal rat CM exposed to OSA (black) or non-OSA (red) sera. (**E**) P65 and (**F**) P50 levels of nucleus lysates prepared from immortalized human CM exposed to OSA (black) or non-OSA (red) sera. Horizontal lines represent median values; the upper and lower box limits indicate the 25th percentile and 75th percentile; whiskers represent the 10th and 90th percentiles.

**Figure 3:**
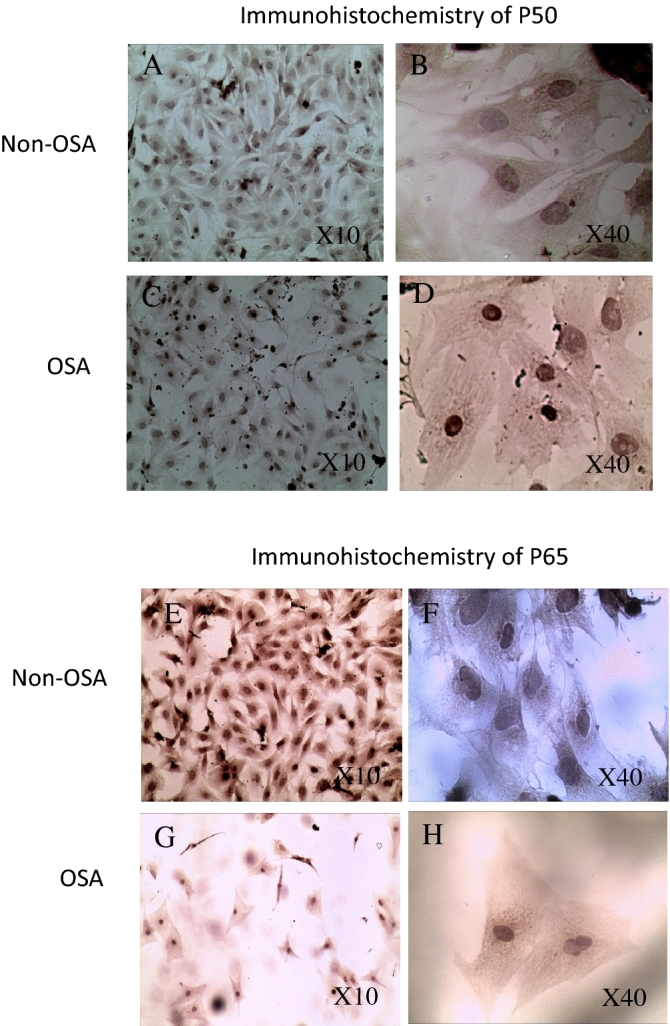
Immunohistochemistry with anti-P50 and anti-P65 staining in neonatal rat CM treated for 48 h. (**A**) Non-OSA serum anti-P50 X100, (**B**) Non-OSA serum anti-P50 ×400, (**C**) OSA serum anti-P50 ×100, (**D**) OSA serum anti-P50 ×400, (**E**) Non-OSA serum anti-P65 ×100, (**F**) Non-OSA serum anti-P65 ×400, (**G**) OSA serum anti-P65 ×100, (**H**) OSA serum anti-P65 ×400.

Further investigation was needed in order to ensure that the increase in P65 and P50 levels we found in OSA serum exposed rat CM translates into NFκB activation. Active NFκB sub-units are disassociated from IκB and may enter the nucleus, where they bind to specific recognition sites in the DNA. We assessed this activation by tracking the translocation of the subunits into the nucleus with two methods: western blot in nuclear lysates (Fig. 2), and immunohistochemistry of P50 and P65 (Fig. 3).

In comparison to samples exposed to non-OSA serum, the samples that were exposed to OSA serum over-expressed P65 and P50 in their nuclear fraction, in western blots.

Immunostaining for NFκB subunits P50 and P65 in neonatal rat CM treated for 48 h showed increased dense staining in the cells' nuclei in CM treated by OSA sera in comparison to those treated by non-OSA serum.

### NFκB pathway is activated by sera of OSA patients

Activation of other players of the NFkB pathway would further confirm its role in cardiac morbidity in OSA patients. Ligation in the suitable receptors on a CM membrane will initiate a phosphorylation cascade. Therefore, we focused on the phosphorylation ratio of IKK proteins, which leads to IKB_α_ phosphorylation and degradation progress by ubiquitin.

**Figure 4:**
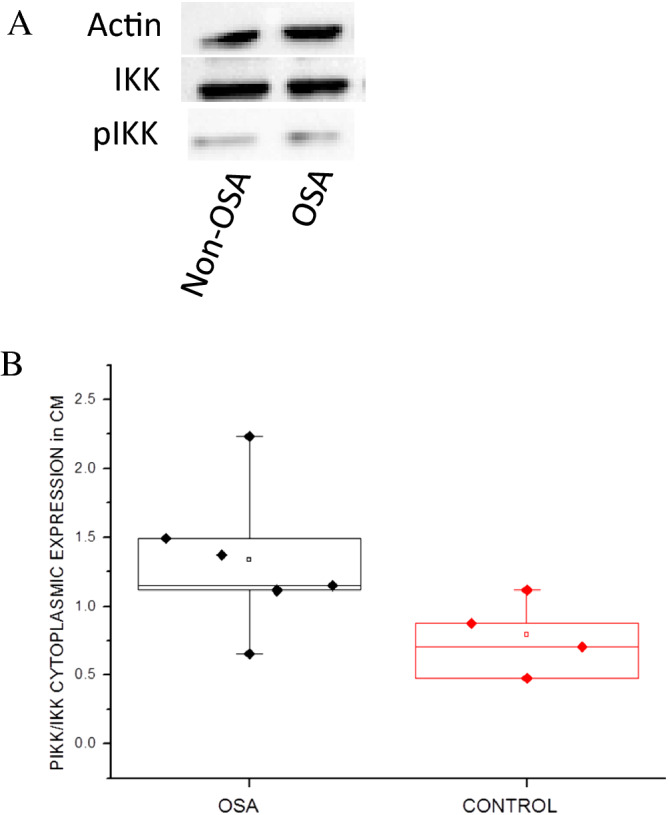
(**A**) Western blot of pIKK and IKK in neonatal rat CM nuclear phase lysate following 2 h exposure to OSA or non-OSA sera. “Non-OSA” refers to 5% Non-OSA patient serum; “OSA” refers to 5% severe OSA patient serum, both in M-119 medium. (**B**) pIKK/IKK ratio levels in nucleus lysates prepared from neonatal rat CM exposed to OSA (black) or non-OSA (red) sera. Horizontal lines represent median values; the upper and lower box limits indicate the 25th percentile and 75th percentile; whiskers represent the 10th and 90th percentiles.

We detected increased phosphorylation ratio levels of IKK in nuclear lysates prepared by a specific protocol from neonatal rat CM exposed to OSA compared to non-OSA sera (Fig. 4).

### Cardiomyocytes morphology is affected by serum of OSA patients

**Figure 5:**
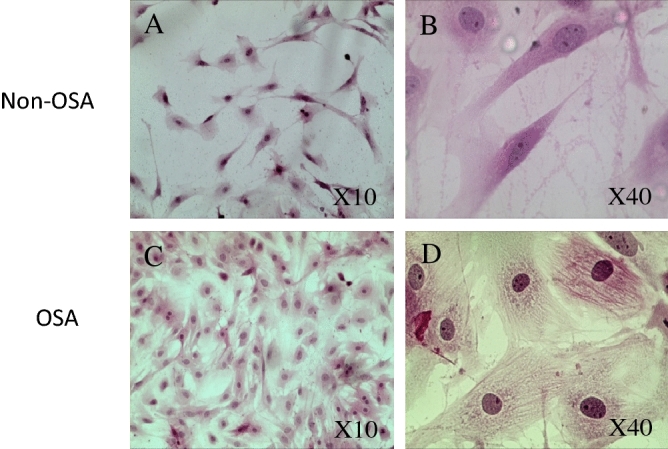
H&E staining of immortalized human CM treated for 48 h with non-OSA serum: [(**A**) ×100, (**B**) ×400]; or with OSA serum [(**C**) ×100, (**D**) ×400].

Hematoxylin and Eosin (H&E) staining allows us to observe CM shape and dimensions more clearly, and enables comparison between OSA-patients and control sera effects on CM morphology. H&E staining demonstrated significant widening and curving of the CM following exposure to OSA sera (Fig. 5).

**Figure 6:**
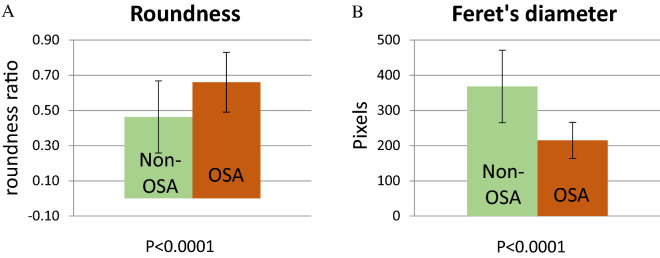
Quantification of morphologic parameters of immortalized human CM cells as analyzed from H&E staining: (**A**) Roundness, (**B**) Feret’s diameter (cell’s elongation). A value of 1.0 in the parameter of roundness indicates a perfect circle (see C.2.6). Feret’s diameter refers to the longest straight line in a certain shape, and represents the degree of elongation of a cell.CM exposed to OSA demonstrated shortening of Feret’s diameter and rounding of the cells (P < 0. 0.0001 in both).

CM exposed to OSA demonstrated shortening of Feret’s diameter and rounding of the cells (Fig. 6, P < 0.0001 in both).

### Cardiomyocytes loss is associated with OSA’s sera exposure

**Figure 7:**
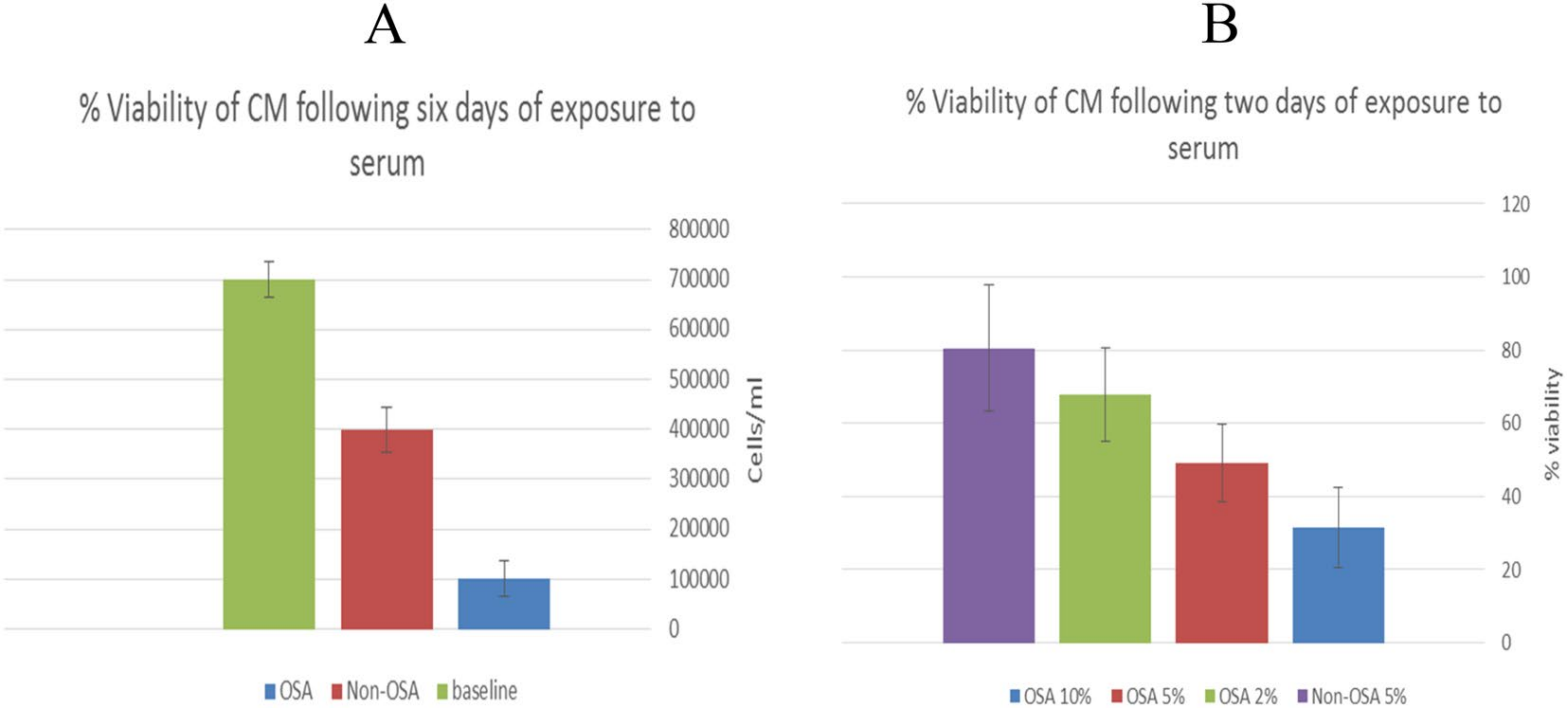
Rat neonatal CM were exposed to FCS (baseline), Non-OSA serum and OSA sera for 6 days, in three consecutive experiments. After 6 days of exposure, dead cells were differentiated with trypan blue, while live cells were counted manually under the microscope using hematocytometer chambers. (**A**) Average live cell count on day 6 of all 3 experiments. (**B**) XTT viability assay following 2 days of exposure to 5% FCS, 5% non-OSA sera, 2%, 5% and 10% OSA sera.

Chronic inflammation could lead to cell programmed death, while CM loss due to multiple mechanisms of death is known to occur in cases of hypertrophy. Supported by morphological changes we found in CM exposed to OSA serum, we further investigated the possible induction of cell death by NFκB in CM. By exposing CM to OSA sera for prolonged periods of time, we assessed their viability and degree of cell loss. Trypan blue and XTT assay kit were used for this purpose (Fig. 7).

## Discussion

In this study, we show how serum of OSA children, activates the NF-κB pathway in several types of CM and causes morphological changes that reflect hypertrophy as well as decreased contractile function. We also identify activation of apoptotic mechanisms, that is not observed following non OSA serum exposure.

OSA occurs in up to 5% of children in the United States. Long-term cardiovascular risks of OSA in adults are well known. Although changes in BP regulation occur in children with OSA, the pathways leading to chronic cardiovascular risks of OSA in children are less clear. The risk factors in adults could carry the same future risk for children^17^.

Systemic inflammation is known to be one of the mechanisms that contribute to end organ damage in patients with OSA. Indeed, CRP is increased in OSA patients^18^. The clinical observation teaches us that conventional treatment in OSA patients like CPAP in adults or tonsillectomy in children decreases not only the AHI, but also systemic inflammation^19,20^. It was also shown that effective treatment, that decreases the AHI is associated with a reduction in systemic inflammation that is significantly associated with an improvement in echocardiographic parameters (a decrease in TR that reflects a decrease in pulmonary pressure)^21^.

So far, no investigations were ever made at the cellular level, but there are several research groups that are carefully investigating the underlying mechanisms that occur in several animal models of OSA.

The intermittent hypoxia (IH) model taught us that rats that are exposed to different protocols of IH develop a decrease in stroke volume left ventricle end diastolic pressure and the cardiac output, and linked the phenomenon with oxidative stress related cellular damage^22^.

Other groups showed that the IH is causing severe endothelial dysfunction, an observation that contributes to our understanding in regard to the development in blood pressure changes that are known in such patients^23^. Changes in endothelial function that were observed in children with OSA, were not seen following tonsillectomy. Importantly, these changes did not occur in children with a family history of heart disease, suggesting a reversible phenomenon in naïve OSA patients^24^.

Our group has previously shown that NF-κB classical pathway derivatives P50 and P65 are over expressed (when compared to controls) in adenoids and tonsils that were surgically removed from OSA patients^25^. We also found elevated activity of NF-κB after OSA serum incubation in two different cell lines. Jurkat cells in which NF-κB is inducible and L428 cells in which NF-κB is constitutively activated^25^.

Encouraged by these findings, we decided to proceed from the site of hypertrophy, where the disease manifests at the clearest presentation in most of the cases, to the end organ, i.e. the cardiomyocytes. We decided to assess the potential systemic effect on several types of CM, because of the known variability between different species in response to the same trigger.

In this work we were able to show that the exposure to patient’s serum is associated with changes in the geometry of the heart at the cellular level.

Changes in the morphology of CM may be the first visible sign for cardiac morbidity which is often characterized by cardiac hypertrophy. CM exhibiting significant concentric hypertrophy present decreased in length and increased in width^26^.

So far, such geometrical changes were described in OSA children^2^ as well as in rats^27^, and our observation may shed some light on one of the underlying mechanisms leading to this phenomenon. The different apoptotic assays we performed showed similar findings, and they coincide with similar findings in rats exposed to intermittent hypoxia^28^. Indeed, a decrease in an anti-apoptotic protein Bcl2 level and Bid and increase of important apoptotic proteins like cytosolic cytochrome c, activated caspase-8, activated caspase-9, and activated caspase-3 were noted in rats exposed to intermittent hypoxia.

The importance of this work is the contribution to the general understanding, that the insult and the damage to CM in OSA patients is at least partially attributed to NFkB related circulating mediators.

With no doubt, other crucial mechanisms like oxidative stress, reactive oxygen species and certainly activation of the autonomic nervous system certainly plays a role in the pathophysiology of CV damage in OSA^29,30^.

Apoptosis of myocardial cells following exposure to apnea was already reported by another group. Almendros et al. reported on apoptosis of myocardial cells in rats exposed to obstructive apneas for 10 days. The rats were subjected to obstructive apneas with a pattern of 15 s each, 60 apneas/h^31^. The investigators also reported significant increase in IL1-β, in similar to the data reported by others^25^.

The novelty of our findings and the contribution of this work is the understanding that there is a major inflammatory pathway that negatively impacts the cardiovascular system of OSA patients. Based on these findings, we intend to explore the effects of NFkB blockers/suppressors on CV parameters in our ex vivo model.

There are several limitations to this work. Although other investigators prefer to use healthy children as controls, we decided to use children with no OSA that are undergoing tonsillectomy (and already getting a venipuncture), and this may effect our data interpretation. We also did not assess serum taken post-surgery to see if the effects we report vanish following surgery, which could have strengthened our conclusions. We did not perform any assays to detect the specific sera component/s that are responsible for the activation of NFkB and other phenomenon we describe. Furthermore, although interesting, there are always differences between in vitro to in vivo studies, and therefore, this work cannot imply clinical practical changes in regard to patients.

## Conclusion

In conclusion, we describe pathological geometrical/functional and biological alterations in cardiomyocyets that were exposed to OSA patient’s sera. We suggest that NFkB is involved in the evolution of cardiovascular damage at the cellular level, and should be looked at as a target for intervention.

## Materials and methods

### Patients and setting

This study was approved by the animal experimental committee of Ben-Gurion University and all methods were carried out in accordance with the relevant guidelines and regulations. The study was also approved by the human IRB/Helsinki committee at the Soroka University Medical Center, and it was carried out in accordance with the relevant guidelines and regulations. Fifteen children, who were previously diagnosed with severe OSA (AHI > 10) during an over-night PSG test were recruited. Fifteen other children were recruited for the control group (non-OSA), when OSA was ruled out at PSG (AHI < 1) (Table 1). The non-OSA children underwent T&A due to recurrent throat infections, and were otherwise healthy. All participants’ parents signed an informed consent form.

All the blood samples were collected from the children in the operating room right before undergoing T&A during the morning hours, and then immediately centrifuged for 5 min at 1200 rpm. Serum fractions were carefully collected and immediately stored at − 80 °C until evaluation. A summary of the demographic information is presented in Table 1. Exclusion criteria were any previous history of cardiovascular disorder, allergies, asthma/wheezing, smoking in the immediate family, and familial craniofacial or genetic disorders.

### Cardiomyocytes models

Three different CM models were used during the study. A. Rat, Neonatal (1–2 days old), sprague dawley, Harlan labs, USA B. immortalized human cardiomyocytes SV40, ABM, USA and C. mouse, 2–3 months old males, C57, Harlan labs, USA.

### Cell cultures

Neonatal rat CM were maintained in M-119 medium. Human immortalized CMs were maintained in Prigrow I medium and were passaged by trypsinization. Both media were supplemented with 5% heat-inactivated fetal calf serum (FCS), 1% l-glutamine, 1% B_12_, 0.2% CuSO_4_**·**5H_2_O + ZnSO_4_**·**7H_2_O, and 1% penicillin–streptomycin. CM were plated on gelatin coated wells or glass slide and kept in 5% CO_2_ atmosphere at 37 °C heated incubator. Adult mouse CM were maintained in suspension for a few hours in Hepes-thyroid buffer (C.1.6).

### Obtaining rat and mouse primary CM

In order to single out and isolate CM from other cell types of the neonatal murine hearts, an ADS digestion buffer was prepared. This buffer contains collagenase type II and pancreatin. After anesthesia with isoflurane, the chest wall was cut open and the heart was isolated by scissors. The ventricles were then cut to small pieces which allow the digestion activity of the enzymes. The digestion phase consists of 6 repetitions of washing, gently stirring and centrifugation of the heart fragments, finally resuspending the pellet with medium and plating the cells.

### Western blot

Neonatal rat CM or human immortalized CM were exposed to different sera for 2 h before lysates were prepared. Whole cell lysates or cytoplasm/nucleus lysates were then studied for the expression of NFκB subunits (P65, P50) and IKK_α/β_ by western blot. All proteins levels were normalized to the lysate’s respective actin levels, than quantified using the densitometry method with the computer program “image J”.

### Immunohystochemistry

Immunohystochemistry was performed with the ABC peroxidase complex method using the Vectastain kit.

Rat neonatal CM were cultured on microscope glass slides, and exposed to different sera for 2 h before being fixed with 98% ethanol. Slides were then incubated with primary antibodies against NFκB classical subunits (P65 or P50, Santa Cruz Biotechnology, Ca, USA) and then with secondary antibodies (Jackson ImmunoResearch, USA) which were conjugated to peroxidase.

### Hematoxilin & Eosin (H&E) staining

Human immortalized CM were cultured on a microscope glass slide and exposed to different sera for 48 h in order to allow morphological changes to occur before being fixed with 98% ethanol. Slides were then stained with hematoxilin and eosin, in order to visualize morphological changes. Hematoxylin has a deep blue-purple color and stains nucleic acids, while eosin is pink and stains proteins nonspecifically.

### Roundness and Feret’s diameter quantification

The computer program “mage J” was selected for photo analysis of H&E stains. A total of 160 stained cells were analyzed and several parameters were quantified. The roundness parameter is calculated by the ratio:

$$\frac{4 \times \text{area}}{\uppi \times {{\text{major}\_\text{axis}}^{2}}}$$

A roundness value of 1.0 indicates a perfect circle.

Feret’s diameter refers to the longest straight line in a certain shape, and represents the degree of elongation of a cell. Normally, CM shape is elongated and has a specific directionality in a tissue, in order to perform efficient cardiac contraction. Shortening of Feret’s diameter and rounding of the cells are morphologic changes that indicate direct implications of cardiac morbidity.

### Trypan blue

Rat neonatal CM were exposed to FCS, non-OSA or OSA sera for 6 days, only then they were trypsinized and mixed with trypan blue. Trypan blue is a dye that can penetrate and selectively stain in blue only cells with porous membrane, usually due to cell death processes. Thus, by looking through a microscope it enables us to distinguish live from dead cells. A hematocytometer is used to manually count the live cells in each sample.

### XTT-viability assay

Rat neonatal CM were exposed to FCS, non-OSA or OSA sera for 48 h, only then tetrazolium salt (XTT) was added (XTT cell proliferation kit: Beit Haemek, Israel). Metabolic active cells reduce the salt with mitochondria enzymes to form the orange-colored formazan. This dye is water soluble which allows specific wavelengths absorbance to be measured with a spectrophotometer. Dye intensity is proportional to the number of metabolic active cells, hence deductive regarding the cells viability.

### Statistical analysis

The comparison between OSA and controls sera effects regarding all experiments was performed using T-Student test. Statistical difference between the groups was considered as significant when P value was under 0.05.

## Supplementary Information

Supplementary Information

## Data Availability

Deidentified individual participant data will not be made available.

## Abbreviations

OSA: Obstructive sleep apnea syndrome
SDB: Sleep disordered breathing
T&A: Adenotonsillectomy
TST: Total sleep time
