# Supplementary material for: Nuclear factor kappa B activation in cardiomyocytes by serum of children with obstructive sleep apnea syndrome
Source: Sci Rep. 2020 Dec 17;10:22115. doi: 10.1038/s41598-020-79187-0 (PMC7747711; doi:10.1038/s41598-020-79187-0)
Supplement: Supplementary file 1 — Supplementary Information [file 41598_2020_79187_MOESM1_ESM.pptx]

## Slide 1
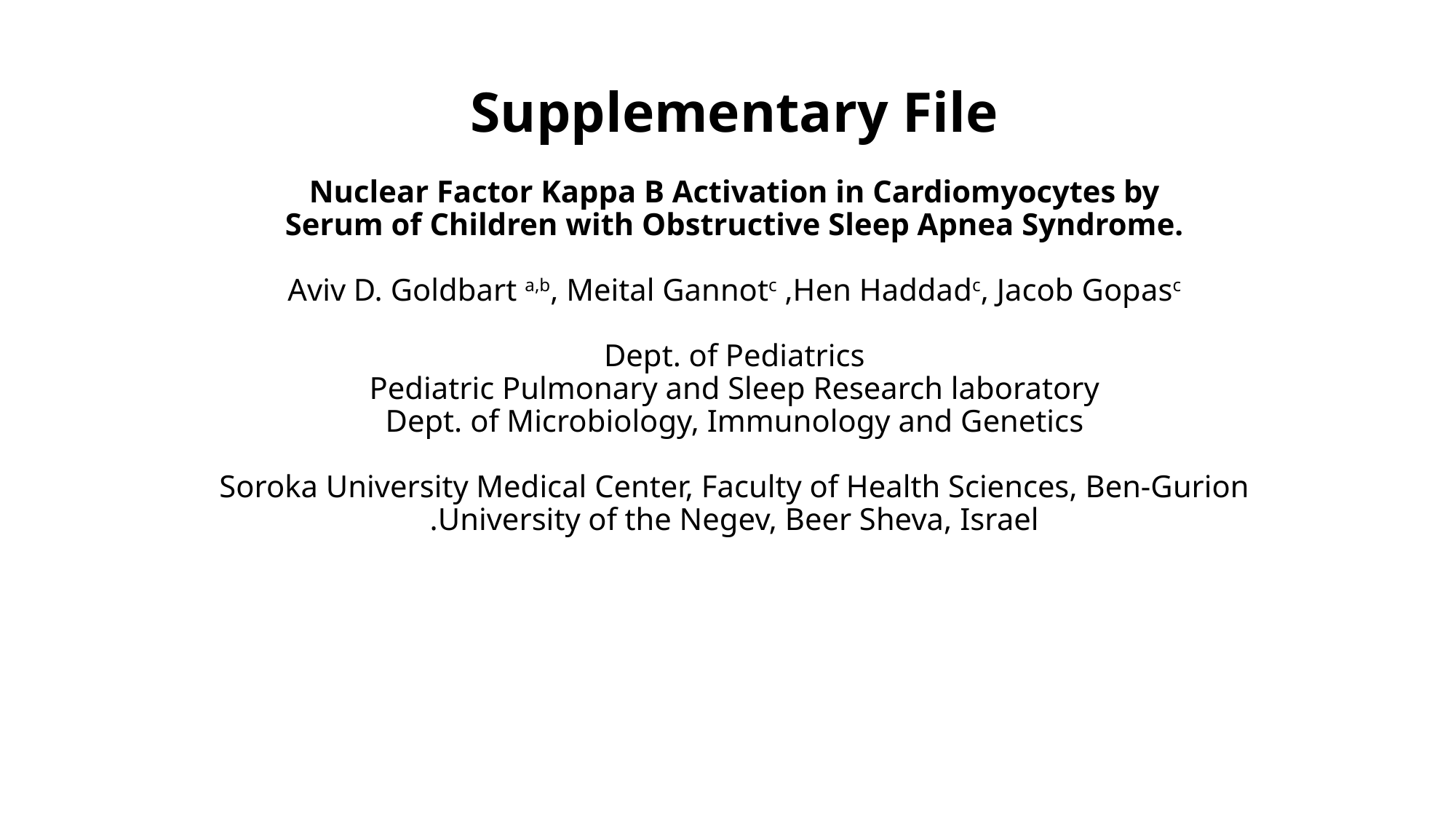

# Supplementary File   Nuclear Factor Kappa B Activation in Cardiomyocytes by Serum of Children with Obstructive Sleep Apnea Syndrome.   Aviv D. Goldbart a,b, Meital Gannotc ,Hen Haddadc, Jacob Gopasc   Dept. of Pediatrics Pediatric Pulmonary and Sleep Research laboratory Dept. of Microbiology, Immunology and Genetics   Soroka University Medical Center, Faculty of Health Sciences, Ben-Gurion University of the Negev, Beer Sheva, Israel.

## Slide 2
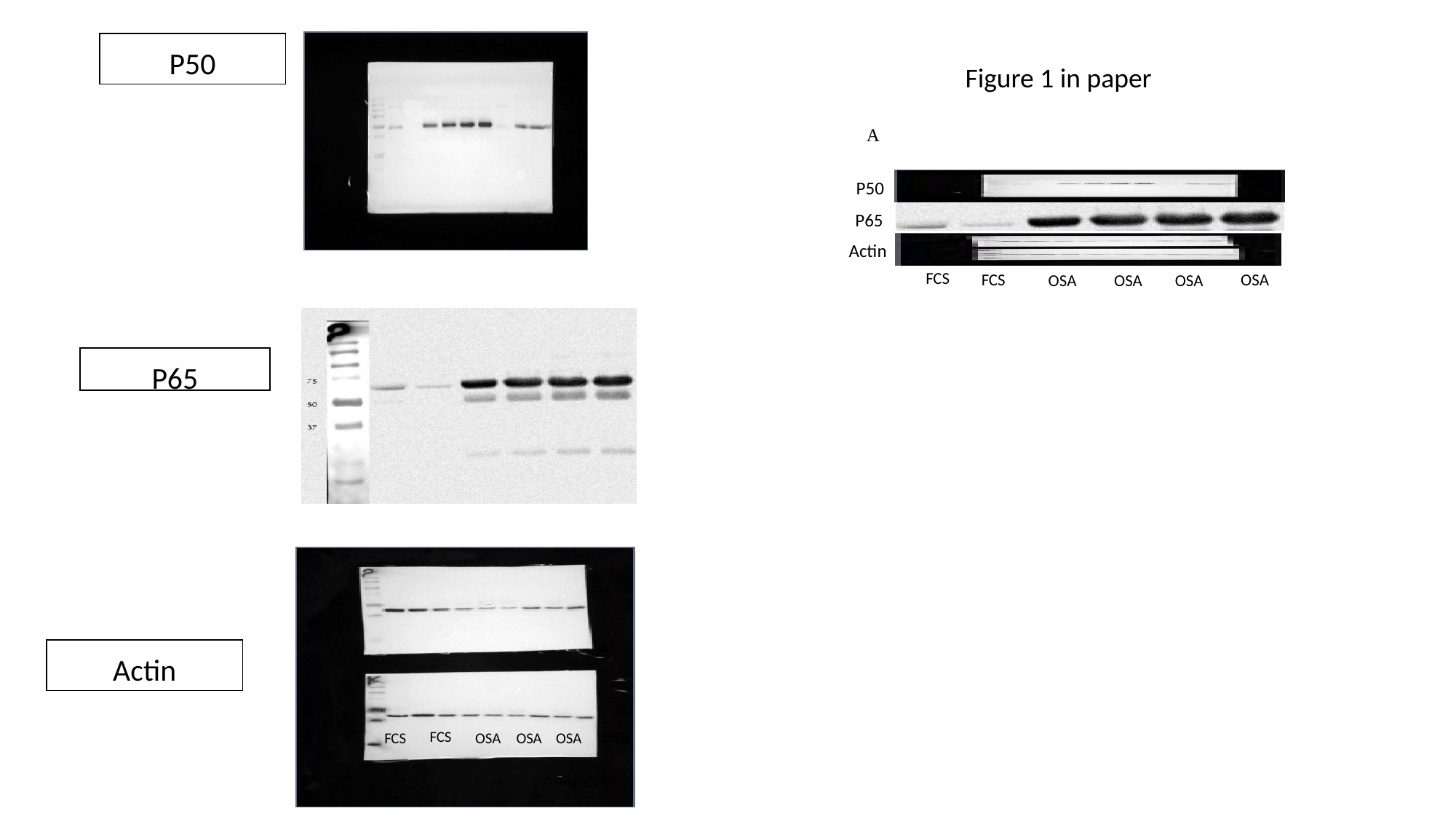

P50
Figure 1 in paper
A
P50
P65
Actin
FCS
FCS
OSA
OSA
OSA
OSA
#
P65
FCS
OSA
OSA
OSA
FCS
OSA
Actin

## Slide 3
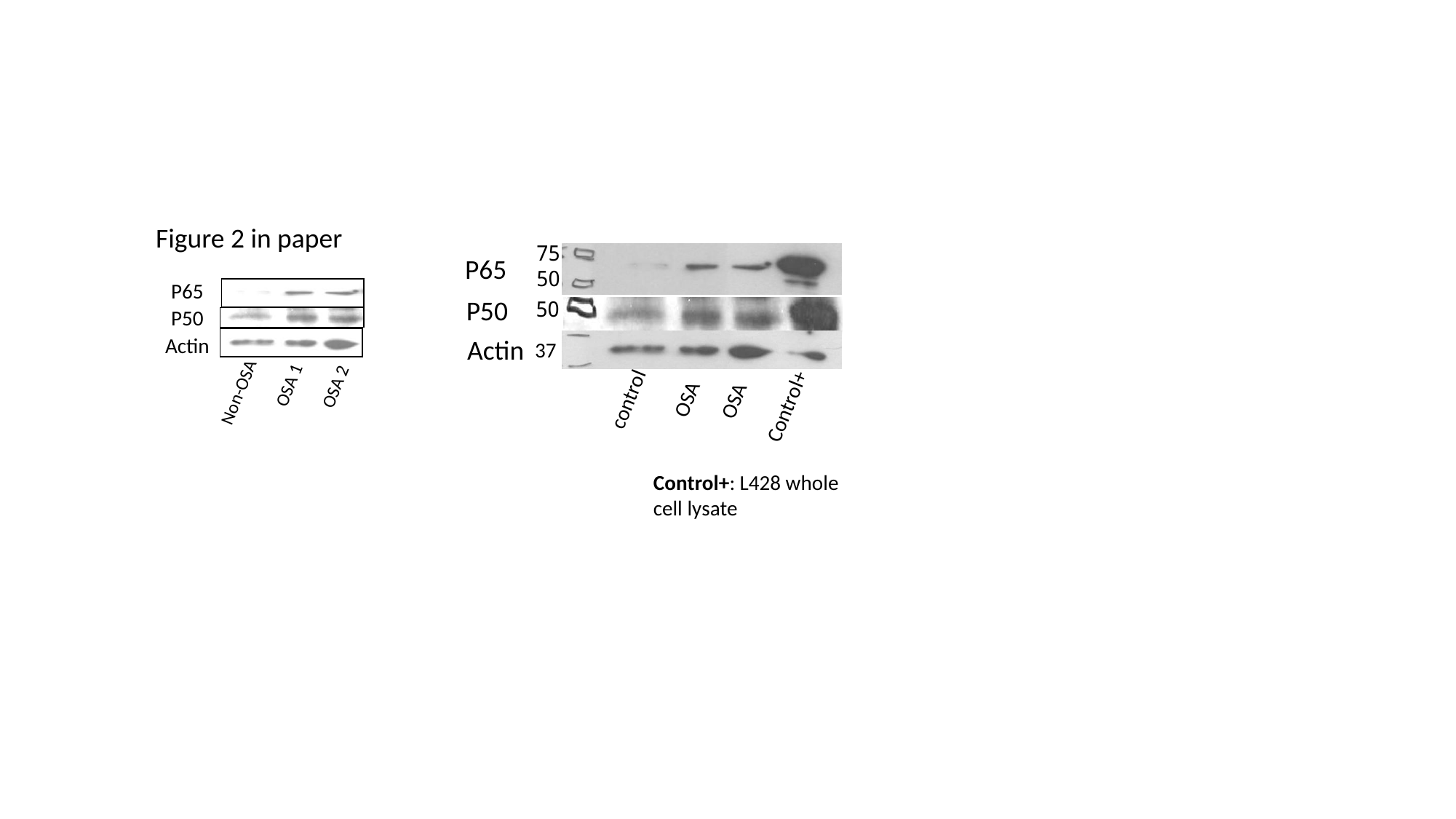

Figure 2 in paper
75
50
P65
P50
50
Actin
37
OSA
control
OSA
Control+
P65
P50
Actin
OSA 1
OSA 2
Non-OSA
Control+: L428 whole cell lysate

## Slide 4
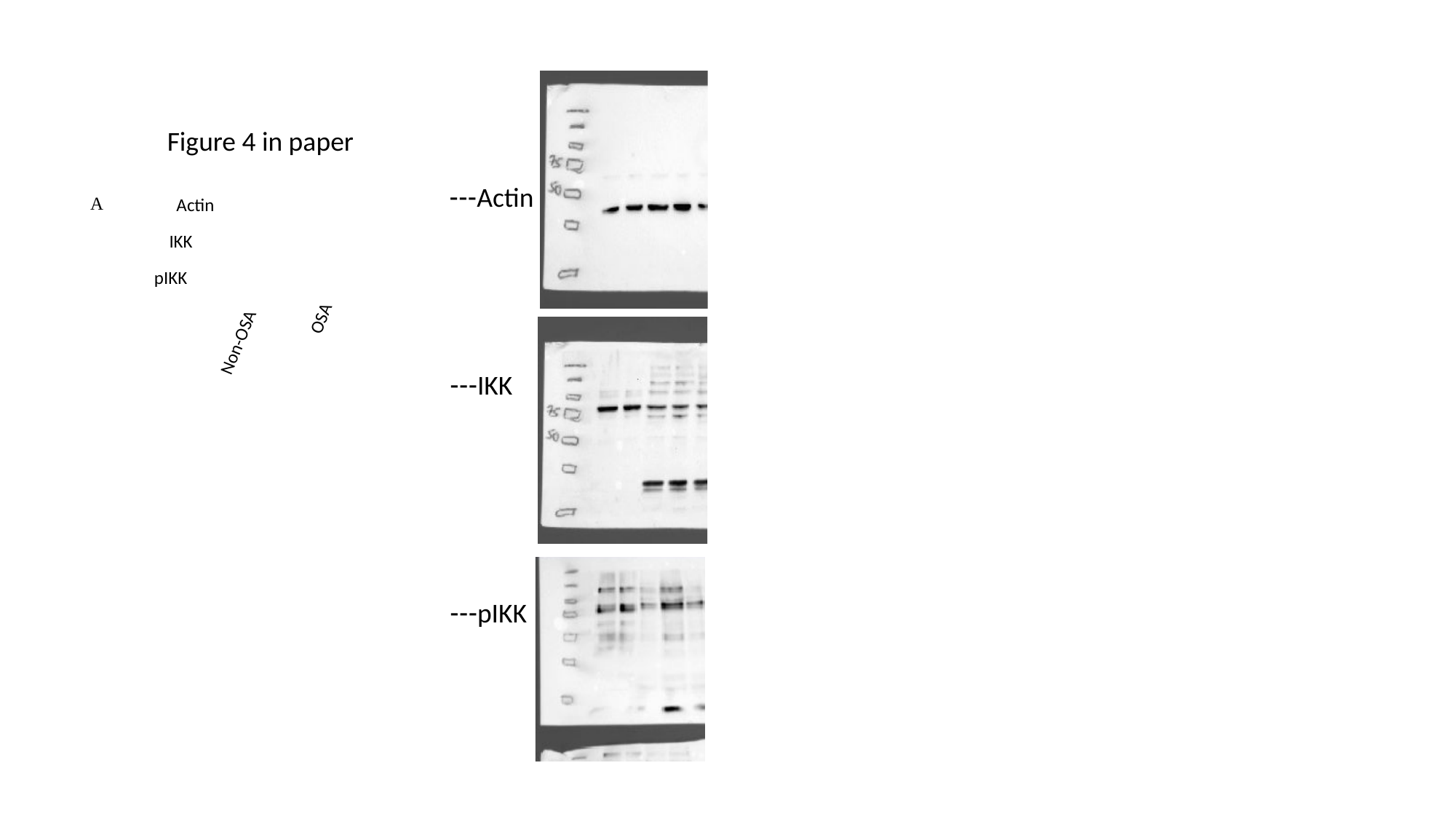

Figure 4 in paper
Actin---
A
Actin
IKK
pIKK
OSA
Non-OSA
 IKK---
pIKK---
